# Developing Central Nervous System and Vulnerability to Platinum Compounds

**DOI:** 10.1155/2011/315418

**Published:** 2011-02-15

**Authors:** G. Bernocchi, M. G. Bottone, V. M. Piccolini, V. Dal Bo, G. Santin, S. A. De Pascali, D. Migoni, F. P. Fanizzi

**Affiliations:** ^1^Laboratorio di Biologia Cellulare e Neurobiologia, Dipartimento di Biologia Animale, Università di Pavia, Via Ferrata 1, 27100 Pavia, Italy; ^2^Istituto di Genetica Molecolare del CNR, Sezione di Istochimica e Citometria, Via Ferrata 1, 27100 Pavia, Italy; ^3^Dipartimento di Scienze e Tecnologie Biologiche ed Ambientali, Università del Salento, 73100 Lecce, Italy

## Abstract

Comparative studies on the effects of the platinum complexes in use or in clinical trials are carried out in order to discover differences in the neurotoxic potential and the reversibility of neurotoxicity. In this paper, we summarized the current literature on neurotoxicity and chemoresistance of cisplatin (cisPt) and discussed our recent efforts on the interference of cisPt and a new platinum compound [Pt(O,O′-acac)(*γ*-acac)(DMS)] (PtAcacDMS), with high specific reactivity with sulphur ligands instead of nucleobases as cisPt, on some crucial events of rat postnatal cerebellum development. The acute effects of drug treatments on cell proliferation and death in the external granular layer and granule cell migration and the late effects on the dendrite growth of Purkinje cells were evaluated. Together with the demonstrated antineoplastic effectiveness *in vitro*, compared with cisPt, data suggest a lower neurotoxicity of PtAcacDMS, in spite of its presence in the brain that involves considerations on the blood brain barrier permeability.

## 1. Introduction

Neurotoxicity of anticancer agents surely complicates treatment of children with cancer. However, it must be taken into consideration that survival rates for children with cancer have increased drastically over the past few decades though chemotherapy in children with hematologic malignancy is associated with elevations of the neurodegenerative marker *tau *in the cerebrospinal fluid, reaching levels observed in other neurodegenerative disorders [1]. In this context, social concerns are increasing for the effects of chemical substances on the nervous system of children. 

 The developing central nervous system (CNS) is much more vulnerable to injury from toxic agents than the adult CNS and prone to impairment [2]. In mammals, there are very sensitive periods of CNS maturation during which the brain regions may be differentially affected by various chemicals [3, 4]. Moreover, as regards rat cerebellum, for instance, it is known that its cytoarchitecture is achieved around twenty days of life, but an insult to cell proliferation affects only some particular cell types forming in its presence [2]. Agents with this action have very different effects in mitotic granule cells and postmitotic neurons, but the reduction of cell number in the internal granular layer (IGL) deeply affects the dendrite growth of Purkinje neurons [5]. This allows many neuroanatomical and functional alterations in the cerebellum as a consequence of postnatal insults [6].

 Exposure or treatment with therapeutic agents induces a variety of functional abnormalities in the brain. Among anticancer drugs, cisplatin (cisPt) is the most common therapy drug (for review, see [7]). It is widely used for childish malignancies as an essential component of multidrug frontline therapy regimens for children [8] with solid tumors (e.g., neuroblastoma). In this regard, it is interesting to note that recent studies have identified a large number of long-term behavioural issues (such as depression, anxiety, and antisocial behavior) in pediatric oncology patients, especially those treated for CNS tumors [9]. The benefit of cisPt is compromised by severe side effects including neurotoxicity [7], that can result in neurodegeneration and cell death (e.g., cerebellum cell loss; [10]). In addition, one of the major obstacles to achieving the desired responses to cisPt treatment is resistance to therapy, for instance, through DNA repair [7]. Unfortunately, some of the targets involved in chemoresistance may also play normal physiological functions, and consequently reversing resistance should be pursued with caution to avoid potential toxicities [11]. For these reasons, several avenues are underway to prevent or minimize the toxicities and conserve the effectiveness of the platinum chemotherapy drugs. One strategy may be the synthesis of new platinum compounds with different target from DNA and low toxicity profile.

 In this paper, we summarized the current literature on neurotoxicity and chemoresistance of platinum compounds and examined our recent efforts on the interference of cisPt and a new platinum compound ([Pt(O,O′-acac)(*γ*-acac)(DMS)])(PtAcacDMS) on some important events of crucial stages of the postnatal cerebellum development [12]. Particular attention was addressed to the acute effects at PD11 (postnatal day 11) of drug treatments on the active cell proliferation in the external granular layer (EGL) and the granule cell migration from the EGL to the IGL and the late effects at PD17 on the tree dendrite growth of Purkinje cells and the regeneration of the EGL. 

### 1.1. Neurotoxicity

The nervous system is frequently the site of symptomatic toxicity of antineoplastic agents. Most common neurological complications involve acute alterations in consciousness, seizures, cerebral strokes, paralysis, neuropathies, leukoencephalopathy, and ototoxicity. In the last years, the expanded use of older agents and the development of new chemotherapeutic compounds had a major impact on the improvement regarding a high-dose chemotherapy, also in connection with stem cell transplantation regimen [13]. The positive results have focused increased attention on posttherapeutic effects of anticancer drugs, but the molecular mechanism of chemotherapy-induced neurotoxicity has not been completely elucidated. 

 Regarding the older agents, comparative studies on the effects of the platinum complexes in use or in clinical trials [7, 14] have been carried out in order to discover differences in the neurotoxic potential and the reversibility of neurotoxicity. CisPt is one of the most effective chemotherapeutic agents that is used in the management of a variety of human tumors, including cancers of the ovary, testis, bladder, lung, cervix, endometrium, and brain [15, 16]. Although a dose-limiting side effect of cisPt has been known to be nephrotoxicity, severe neurotoxic effects have also been noted [7, 17]. Implications for clinical neuropsychology arising from neurological disruption associated with cisPt-based therapy have been reported [18]. 

 Platinum drugs appear to affect the axons, myelin sheath, neuronal cell bodies, and the glial structures of the neural tissue [19]. At the cellular level, the chemotherapy interferes with DNA replication and metabolic function of the neurons [20]. Platinum-based agents have the propensity to enter the dorsal root ganglia and peripheral nerves [21] as opposed to the brain [22].

 In the last years, several morphological and molecular changes have demonstrated the vulnerability to a single injection of cisPt during the postnatally developing cerebellum, including the degeneration of proliferating and postmitotic neurons. Numerous haemorrhagic foci were found in the whole cerebellar cortex, mainly seven days after treatment performed at postnatal day 10 [23]. More recently, apoptotic cell death in the EGL and concomitant alteration of migratory process was clearly detected [12]. These findings may represent factors influencing the Purkinje cell differentiation and formation of synaptic contacts within the cerebellum neural circuits. In the studies of the effects of cisPt on neurotransmitter molecules involved in the brain maturation, we demonstrated that GABA, glutamate, and monoamines are involved in the developmental cisPt-induced changes of cerebellum architecture [23–26]. However, repair and reorganization with remodelling of the cerebellar cortex cytoarchitecture occurred in the developing cerebellum after the acute cisPt-induced injuries. They include recover of proliferation activity in the EGL, granule cell ectopia in the molecular layer (ML), compartimentation of the ML linked to reorientation of Purkinje cell dendrite branches, and changed distribution of GABA-positive inhibitory interneurons [26] and VGluT2 positive-climbing fibre synaptic contacts [27]. 

### 1.2. Chemoresistance

CisPt is a potent chemotherapic drug and has a major impact in cancer medicine. The benefit of this drug is limited by neurotoxicity and resistance. In general, cisPt is a multifactorial agent; in fact, its biochemical mechanism depends on the interaction of more intercellular changes [28], such as decreased intracellular drug accumulation, rapid metabolism, or excretion, activation of oncogenes and inactivation of tumor suppress genes, alternations in the cell cycle and checkpoints, alternations in apoptotic pathways, altered accumulation of drugs within cells, increased detoxification mediated system of glutathione S-transferase (GST), increased DNA repair, and insensitivity to apoptosis [29, 30]. DNA is the primary target of platinum, and DNA repair can mediate a mechanism of drug resistance, blocking apoptosis. Several factors modulate and contribute to the increase of DNA damage repair. For example, NER, nucleotide excision repair, is the major pathway for platinum adduct removal and DNA repair [31]; Furuta and collaborators in 2002 [32] have shown that cells deficient specifically in NER are hypersensitive of cisPt. GSH (glutathione) is another cisPt target; cisPt resistant cells often show high GSH levels [33]. High GST levels are correlated with cisPt resistance in ovarian cancer cells [34] and other tumors [35]. Another factor involved in the process of resistance is represented by the microtubule alterations [36]; defective formation of the endocytic recycling compartment may lead to cisPt resistance and reduce uptake of cisPt and other molecules [37]. Resistance has also been associated with abnormal sorting of some lysosomal proteins, with cisPt transporters into an exosomal pathway [38], with drug sequestration in subcellular organelles such as melanosomes, with significantly reduced drug nuclear localization and with increased extracellular transport of melanosomes containing cisPt [39]. Finally, the combination of all these factors responsible for drug resistance leads to the inhibition of apoptosis, both *in vivo* and *in vitro*, in different types of cancers. Apoptosis can be initiated either by activation of death receptors on the cell surface membranes (extrinsic pathway) or through a series of cellular events primarily processed by mitochondria (intrinsic pathway). Defects in apoptosis can result in the expansion of a population of neoplastic cells. The molecular changes, that have the potential to cause apoptotic dysregulation, include activation of antiapoptotic factors (Bcl2, BclX_L_, Bfl1/A1, etc.), inactivation of proapoptotic effectors (p53, p53 pathway), and/or reinforcement of survival signals (Survivin, FLIP, NF-*κ*B, etc.). Recently, it has been shown that cisPt induces apoptosis by intrinsic pattern in neuroblastoma rat cells [36], with increased permeability membrane, decreased expression of Bcl2, and Bax translocation into mitochondria [40]. About drug resistance, the mitochondrial membrane undergoes permeability changes [41]; there is an increase of Bcl2 antiapoptotic factor [42]. There are evidences that drug resistance results in induction of cell death through autophagic pathway [43]. Autophagy is a type of cellular catabolic degradation response to nutrient starvation or metabolic stress; it has important roles in developing tissues and in the control of neoplastic potential. Recently, Chen and Debnath [44] have demonstrated that autophagy represents an important survival mechanism for cancer cells during anticancer therapies. High levels of autophagy, observed in tumor cells after anticancer treatment, are commonly an adaptive response that enables tumor cells to survive the therapeutic insult and then to develop drug resistance [45]. Santin and collaborators [41] show that cisPt is able to activate this mechanism to induce drug resistance.

 Muscella and collaborators show that new compounds of platinum PtAcacDMS may induce cell death without binding DNA [46]. This mechanism of action could allow us to overcome toxicity and drug-resistance phenomena,s and it may represent a new therapeutic treatment against tumor. 

### 1.3. The New Complex of Platinum

Current researches have shown insight into the mechanisms of nerve damages caused by platinum agents [47, 48]. Apoptosis has been observed in the neurons of dorsal root ganglion following cisPt treatment both *in vitro* and *in vivo* [49]; it is correlated with intracellular accumulation and increased formation of DNA adducts. Although the mechanism of the transition from a platinum-DNA adduct to neuronal apoptosis is not fully understood, one proposal has suggested that the DNA repair machinery is unable to repair the damaged DNA [50]. It has also been proposed that the cisPt adducts may interfere with cell metabolism [23, 51]. Among the strategies to overcome toxicity and resistance problems, related to the clinical use of cisPt, it should be mentioned out the synthesis of new platinum compounds with different target from DNA and low toxicity profile. Interesting biological properties have been shown by a class of new platinum complexes containing acetylacetonate (Acac) and sulfur ligands such as dimethylsulphoxide (DMSO) or dimethylsulphide (DMS) in the platinum coordination sphere [52]. In particular, the complex PtAcacDMS containing two Acac (one O,O′-chelate, one sigma linked by methine in gamma position) and dimethylsulphide (DMS) in the metal coordination sphere not only was able to induce apoptosis in endometrial cancer cells (HeLa), with activity up to about 100 times higher than that of cisPt, but also showed high cytotoxicity in cisPt resistant MCF-7 breast cancer cells [46, 53].

 The cytotoxicity of the new compound PtAcacDMS is related only to its intracellular accumulation [46, 53]. The new complex, as well as its specific biological activity, showed an interesting and selective chemical reactivity against nucleophiles with different HSAB (Hard-Soft-Acid-Base) character, even in the case of biological molecules such as nucleic acid bases (guanosine, 5′-GMP) and sulphur amino acids (methionine) [53]. The low reactivity with nucleobases and the specific reactivity with sulphur ligands suggest that the cellular targets could be aminoacid residues in proteins and enzymes involved in apoptotic induction. Moreover, mutagenic tests (Salmonella *his-*reversion test, Ames' Test, a standard reverse mutation assay on the mutagenic capability of the complexes) were performed on PtAacDMS using cisPt as a positive control. Interestingly, whereas cisPt exhibited the well-known mutagenic activity, the new complex did not show the presence of revertant colonies, thus confirming that the biological activity of the new Pt(II) complex is related to the reaction with nongenomic biological targets [54]. These results, together with a study on intracellular signal transduction, triggered by the Pt(II) complex, indicate that DNA is not the main target for this complex, characterized by low reactivity with nucleobases and specific reactivity with sulphur ligands. 

## 2. Experimental Schedule for the Treatment with Platinum Compounds

Ten-day-old Wistar rats were given a single subcutaneous injection in the nape of the neck at a dose of 5 **μ**g/g b.w. (corresponding to the therapeutic dose suggested by Bodenner et al. [55]) or 10 **μ**g/g b.w. [56] of cisPt (0.5 mg/mL; Teva Pharma, Italy) or the new compound PtAcacDMS. The chemical structure [52, 54] of the complex PtAcacDMS is as follows. 

 Throughout the experiment, the rats were kept in an artificial 12 h light:12 h dark cycle and provided rat chow and tap water *ad libitum*.

 One day (PD11) and 7 days (PD17) after drug administration, treated and untreated control rats of the same age were deeply anesthetized with an intraperitoneal injection of 35% chloral hydrate (100 *μ*L/100 g b.w.; Sigma, St. Louis, MO, USA). The brains were quickly removed, fixed in Carnoy's solution (6 absolute ethanol: 3 chloroform: 1 glacial acetic acid) for 48 h, then placed in absolute ethanol and in acetone, and embedded in Paraplast X-tra (Sigma). Paraplast-embedded sections of cerebellar vermis were obtained in the sagittal plane. 

 All experiments were performed according to the guidelines for care and use of laboratory animals as published by the Italian Ministry of Health (DDL 116/92). All efforts were made to minimize the number of animals used and their suffering. 

### 2.1. Light Microscopy Immunohistochemistry

Slides with Paraplast-embedded sections were dewaxed in xylene, rehydrated in a decreasing ethanol series, rinsed in distilled water, and immunostained as described below. 

#### 2.1.1. Bax, Bcl2, and GAD67 Immunoreactions

The endogenous peroxidases were suppressed by incubation of sections with 3% H_2_O_2_ in 10% methanol in phosphate-buffered saline (PBS; Sigma, St. Louis, MO) for 7 min. Subsequently, the sections were incubated for 20 min in normal serum at room temperature in order to block nonspecific antigen-binding sites. Localization of Bax, Bcl2, and GAD67 (Glutamic Acid Decarboxylase) was achieved by applying on brain sections, respectively, a rabbit polyclonal anti-Bax (1 : 30; Santa Cruz Biotechnology, Santa Cruz, CA), a rabbit polyclonal anti-Bcl2 (1 : 20; Santa Cruz Biotechnology, Santa Cruz, CA), and a rabbit monoclonal anti-GAD67 (1 : 200; Santa Cruz Biotechnology, Santa Cruz, CA) in PBS overnight in a dark moist chamber. Thereafter, the sections were sequentially incubated with biotinylated secondary antibodies (1 : 200; Vector Laboratories, Burlingame, CA, USA) for 30 min and horseradish peroxidase conjugated avidin-biotin complex (Vector Laboratories, Burlingame, CA) for 30 min at room temperature. Then, 0.05% 3,3′-diaminobenzidine tetrahydrochloride (DAB; Sigma, St. Louis, MO) with 0.01% H_2_O_2_ in Tris-HCl buffer (0.05 M, pH 7.6) was used as a chromogen. After each reaction step, sections were washed thoroughly in PBS (two changes of 5 min each). Sections were dehydrated in ethanol, cleared in xylene, and mounted in Eukitt (Kindler, Freiburg, Germany). The slides were observed with an Olympus BX51 microscope, and the images were recorded with an Olympus Camedia C-5050 digital camera and stored on a PC. Corrections to brightness and contrast, as well as the conversion of colour images to gray-scale images, were made with Paint Shop Pro 7 (Jasc Software Inc). 

 For control staining, some sections were incubated with PBS instead of the primary antibody. No immunoreactivity was present in this condition. 

### 2.2. Fluorescence Microscopy Immunohistochemistry

#### 2.2.1. Calbindin

The immunoreaction was performed overnight at room temperature using a primary mouse monoclonal antibody anti-CB (1 : 5000, Swant, Bellinzona, Switzerland) diluted in PBS. Sections were washed in PBS and incubated with the secondary antibodies Alexa-Fluor 488 goat antimouse (1 : 100, Molecular Probes, Space, Milan, Italy) in PBS for 1 h. After washing in PBS, the nuclei were counterstained with 0.1 *μ*g/mL Hoechst 33258 for 6 min, and coverslips were lastly mounted in a drop of Mowiol (Calbiochem, San Diego, CA, USA). The slides were viewed by fluorescence microscopy with an Olympus BX51 equipped with a 100 W mercury lamp used under the following conditions: 330–385 nm excitation filter (excf), 400-nm dichroic mirror (dm), and 420-nm barrier filter (bf), for Hoechst 33258 and 450–480 nm excf, 500 nm dm, and 515 nm bf for Alexa 488. Images were recorded with an Olympus Camedia C-5050 digital camera and stored on a PC. Images were optimized for color, brightness, and contrast by using Paint Shop Pro 7 software (Jasc Software Inc). 

 For control staining, some sections were incubated with PBS instead of the primary antibodies. No immunoreactivity was present in these sections. 

### 2.3. TUNEL Histochemical Reaction

The reaction was performed using a TdT-mediated dUTP nick end-labelling kit (Roche, Penzberg, Germany).

 The sections were dewaxed in xylene, rehydrated in a decreasing ethanol series, rinsed in PBS (Sigma), and incubated with a permeabilization solution (0.1% Triton X-100, 0.1% sodium citrate in PBS) for 8 min at room temperature. After rinsing two times in PBS, slides were incubated with 50 *μ*l of TUNEL mixture conjugated with FITC (5 **μ**L terminal deoxynucleotidyl transferase solution and 45 **μ**L label solution) according to the manufacturer's instructions for 3 h at 37°C. The sections were rinsed in PBS, and the cells were counterstained with 0.1% trypan blue in PBS. After washing with PBS, the nuclei were counterstained with 0.1 *μ*g/mL Hoechst 33258 for 5 min, and coverslips were mounted in a drop of Mowiol (Calbiochem, San Diego, CA, USA). 

 The slides were observed by fluorescence microscopy with an Olympus BX51 microscope equipped with a 100 W mercury lamp used under the following conditions: 330–385 nm excitation filter (excf), 400-nm dichroic mirror (dm), and 420-nm barrier filter (bf), for Hoechst 33258; 450–480 nm excf, 500-nm dm, and 515-nm bf, for FITC. Images were recorded with an Olympus Camedia C-5050 digital camera and stored on a PC using Olympus software, for processing and printing.

 For the control staining, some sections were incubated without enzyme solution. No reactivity was observed. 

## 3. Results and Discussion

The observations are performed on the apical zones of VI to VIII cerebellar vermis lobules, since these parts of cerebellum show still an active proliferation and differentiation [5] at 10 days of postnatal life, that is, the date we injected rats with the platinum compounds. 

### 3.1. Cell Proliferation, Death, and Migration

The complex layered architecture of cerebellum, like the cerebral cortex and hippocampus, adds cells over a long periods and cell production creates numbers of neurons in excess of the number actually needed [5, 57]. The overproduction is normally followed by a wave of cell death (programmed cell death) to establish the proper final number of neurons [58, 59]. As regards the postnatally developing cerebellum, a germinative matrix (EGL) persists until the third week [5]. The matrix originate the granule cells only [60] of the IGL, after their migration through Bergmann radial glia fibres. It is worthwhile that cerebellar defects were also found to result from a failure of Bergmann radial glia [61], that represents a mechanical force of cerebellar foliation [62, 63] for the achievement of the normal cerebellum architecture. The strict relationship between altered proliferation and damaged migration has been also demonstrated with X-ray irradiation [64], MAM [64, 65], or methylmercury [66–68].

 Paying attention to the programmed cell death, that has a critical role during development and in normal cellular homeostasis, it has been equated with apoptosis, although nonapoptotic forms of cell death also exist, for example, autophagy [69]. The TUNEL reaction is a known marker for cell apoptosis, which is regulated by the prototypical members of a large family of Bcl2-like proteins such as Bax and Bcl2 (for review, see [70]). A major role in regulating neuronal death in the CNS was assumed by the proapoptotic molecule Bax, during development and after injury [71, 72]. Bcl2 is an antiapoptotic protein expressed at high level during development of CNS and downregulated after birth; Bcl2 is crucial in the maintenance of neuronal survival [70].

**Figure 1:**
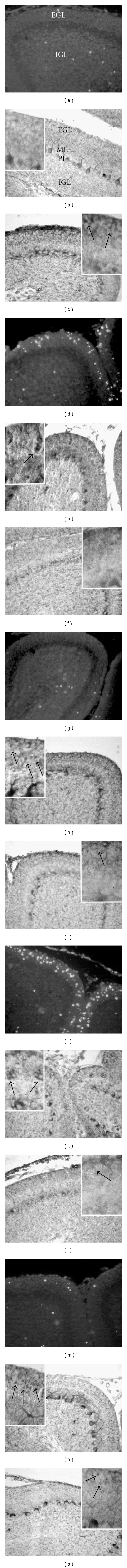
Cell death and damage of proliferating and differentiating cells at PD11. *TUNEL reaction, Bax and Bcl2 immunoreactions*. *Treatment with dose of 5 *μ*g/g b.w.* At PD11, in comparison with control rats (a)–(c), TUNEL positive dying cells increase in the EGL after cisPt (d) as well as Bax immunopositivity (e), especially in nests of cells (e, insert: arrow), while Bcl2 labelling decreases (f); in the Purkinje cell bodies, the immunopositivity for Bax is high (e), while Bcl2 is lowered (f). After PtAcacDMS, there are no changes of TUNEL reactivity (g); Bax-labelled (h) microglial-like cells (h, insert: arrows) are more distinguishable in the EGL, and Bax immunopositivity increases in the Purkinje cells (h); the labelling for Bcl2 (i) is present in some proliferating cells of the EGL (I, insert: arrow). *Treatment with dose of 10 *μ*g/g b.w.* There is a further increase of dying cells after cisPt (j) in the EGL, where nests of Bax immunopositive cells (k) and Bcl2 immunonegative cells (l inserts: arrows) are evident; weak Bcl2 immunopositivity is shown in the EGL and in the Purkinje cells (l, insert: arrow). In comparison with control rats (a), no changes are observed after PtAcacDMS regards TUNEL reaction (m), but several Bax immunopositive microglial-like cells (n) are detectable in the EGL (insert: arrows) as well as some Bcl2 cells (o, insert: arrows); there is an increased Bcl2 immunoreactivity in the Purkinje cell bodies (o). Magnification: 40x; insert: 100x.

**Figure 2:**
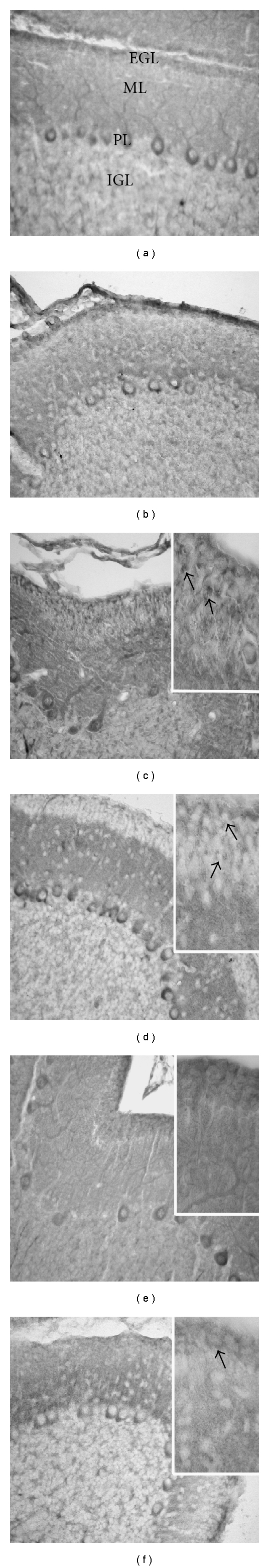
Cell death and damage of proliferating and differentiating cells at PD17. *Bax and Bcl2 immunoreactions. Treatment with dose of 10 *μ*g/g b.w.* In comparison with control rats (a, b), an increased labelling for Bax is observed after cisPt (c). Several microglial-like cells, that are labelled for Bax (c, insert: arrows) and Bcl2 (d, insert: arrows), are present in the EGL. The immunoreactivity for Bcl2 in the Purkinje cell bodies (d) is intense. After treatment with PtAcacDMS, Bax (e) and Bcl2 (f) immunoreactivities are similar as in control rats; in particular, some Bcl2 proliferating cells (f, insert: arrows) are detectable in the EGL. Magnification: 40x; insert: 100x.

 CisPt exerts its neurotoxic action on proliferating cells leading to a dramatic death of immature granule cells of the EGL at early developmental stages [12]. This decrease is to a large extent attributable to induction of apoptosis. Findings here shown (Figures 1(a)–1(o) indicate that, comparing with control rats (Figure 1(a)), cell death was higher at PD11 with cisPt (Figures 1(d), and 1(j) and confirm that cisPt effects are dosedependent [73, 74]. On the contrary, PtAcacDMS at both the doses (Figures 1(g), and 1(m)) did not alter the distribution and labelling for TUNEL reaction. Moreover, comparing with the controls (Figures 1(b) and 1(c)) there were evident changes of Bax (Figures 1(e), 1(h), 1(k), and 1(n)) and Bcl2 (Figures 1(f), 1(i), 1(l), and 1(o)) immunoreactivities after platinum compound treatments in the EGL and Purkinje cell layer. As previously reported [74], no changes in TUNEL labelling for both the doses of the cisPt and PtAcacDMS were found at PD17 (not shown), differently from Bax (Figures 2(c) and 2(e)) and Bcl2 (Figures 2(d) and 2(f)) immunoreactivities, in respect of the controls (Figures 2(a) and 2(b)).

 In particular, at PD11, comparing with control rats (Figures 1(b) and 1(c)), immunoreactivity for Bax in the immature neurons of the EGL was observed after cisPt treatment (Figures 1(e) and 1(k)); the high dose revealed nests of cells that are Bax intensely labelled (Figure 1(k)) and Bcl2 immunonegative (Figure 1(l)). After PtAcacDMS (Figures 1(h) and 1(n)), there was increased immunoreactivity for Bax in very small cell elements ascribable to microglial-like cells. In both the platinum compound treatments, Bax immunoreactivity of Purkinje cells was more intense than in controls. Conversely, the labelling for Bcl2 was lowered after cisPt in the EGL and Purkinje cells but maintained the steady state after PtAcacDMS at 5 *μ*g dose and increased in the Purkinje cells at 10 *μ*g dose. Hence, differently from cisPt, PtAcacDMS counterbalanced early the increased Bax immunoreactivity maintaining the Bcl2 steady state of controls, especially in the EGL after the single dose, or increasing the labelling in the Purkinje cells. This balance might assure a limited cell death in the EGL germinative layer and cell degeneration in the Purkinje cell population. Not only the Purkinje neurons had an almost normal differentiation (see Section 3.2) in PtAcacDMS-treated rats, but also a delay, followed by alteration, of the dendrite growth occurred after cisPt. 

 Interesting findings were obtained at 7 days after treatments, when at the high dose regeneration of EGL occurs [74]. Two different patterns were found: (1) after cisPt, the recovery of EGL thickness [74] displayed higher presence of Bax-labelled microglial-like cells (Figure 2(c)) and Bcl2 (Figure 2(d)) labelling in the proliferating cells of the EGL, (2). after PtAcacDMS, the intensity and distribution of immunoreactivities were similar as in controls (Figures 2(a) and 2(b)), both for Bax (Figure 2(e)) and Bcl2 (Figure 2(f)).

 Hence, we provide evidence that, following a drastic cell death at PD11, the EGL may regenerate after cisPt, but the delay in the increased immunoreactivity for Bcl2 antiapoptotic protein can induce, like a cascade, altered dendrite growth of Purkinje cells and synaptogenesis, and then altered cytoarchitecture [26, 27]. 

 In this study, the presence of microglial-like cells in all the cerebellar layers merits attention. During development, microglia contribute to the reorganization of neuronal connections, although microglia have also pivotal roles during acute and chronic neurodegeneration and are found to be activated under several pathological conditions or neurodegenerative processes (for a review, see [75]). Microglial cells have populated the developing CNS before the neuronal loss occurs and are involved in the removal of cellular debris and dead cell corpuses [76].

 Massive invasion of microglia was previously described by one of us [73] in some regions of cerebellar cortex at 10–15 postinjection stages of 5–10 *μ*g cisPt. The invasion was related to the presence of hemorrhagic foci and the damages of small blood vessels [23]. Here, the labelling for Bax and Bcl2 showed microglia-like cells in the EGL of controls and treated rats. Platinum compounds seemed to induce an increase of Bax proapoptosis protein, but the immunoreactivity for Bcl2 was unchanged. The changed balance Bax/Bcl2 can lead to subsequent microglial apoptotic cell death, as shown recently after interferon gamma treatment [77]. Based on these data, microglia apoptosis should occur at early stage after treatment with PtAcacDMS, while it should characterise the late stage (PD17) after cisPt. The undergoing cell death of microglia is difficult to explain considering the role of microglia and the fact that mainly after PtAcacDMS a low number of TUNEL apoptotic cells were found. On the other hand, microglia activated by ischemic insult through an upregulation of proapoptosis proteins may increase reactive oxygen species generation, resulting in neuronal injury [78]. The intriguing findings we have obtained are still far away from elucidation and need further studies with specific markers [79].

 Moreover, vulnerability was not restricted to dividing cells since glial cells (e.g., Bergmann radial glia) and postmitotic neurons (e.g., Purkinje cells) were also directly damaged by this drug as noted in literature [24, 26, 80–82]. The altered immunoreactivity for GFAP and morphology of Bergmann radial glia fit well with the decreased thickness of the EGL premigratory zone at early stage. On the other hand, it cannot be excluded that cisPt exerts an indirect action on some of the differentiating cerebellar cell populations. In fact, it is known that there exists a close relationship among granule cell precursors, glial fibres, and growth of Purkinje cells dendritic tree (for review, see [62]). Moreover, it is necessary to consider that cisPt could act not only on DNA but also on proteins [83], cytoplasmic components (e.g., mithocondria, cytoplasmic reticulum), and metabolic enzymes [51, 84] broadening the sphere of cell types on which it exerts its cytotoxic action. Apart from the different action, PtAcacDMS did not damage the glial fibres and consequently should not alter cell migration [74]. 

### 3.2. Cell Differentiation and Synaptogenesis

The maturation of the GABAergic Purkinje neurons occurs postnatally in the cerebellum [5]. These neurons are the only output and act as final decoders of information from the cerebellar cortex. They form synaptic contacts with parallel and climbing fibres, and the axons of ML interneurons and Golgi neurons. Alterations of these contacts affect the growth of Purkinje cell, especially of its dendrite (for review, see [26, 27]. Degeneration of 20% of Purkinje cell population has been described after cisPt treatment [23].

**Figure 3:**
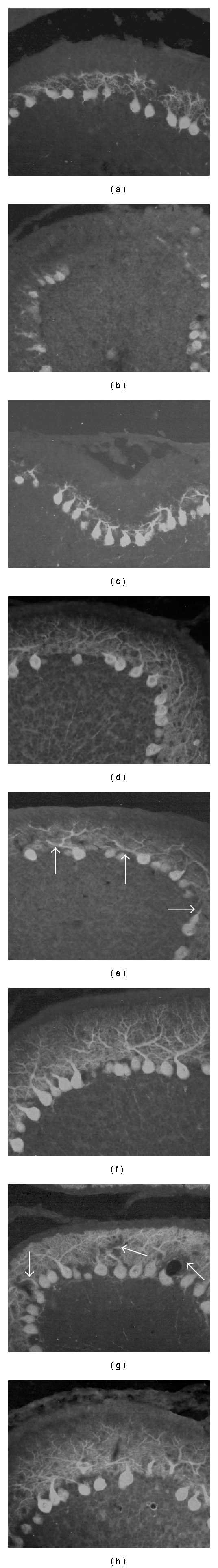
Purkinje cell dendrite growth: calbindin fluorescence immunoreaction.* Treatment with dose of 5 μg/g b.w.* At PD11, compared to control rats (a), the dendrite of Purkinje cells appears less extended in the cisPt-treated rats (b); some tracts of lobules lack Purkinje cells (b). After treatment with PtAcacDMS, a less marked delay in branching is shown (c). At PD17, in respect of control features (d), the main dendrite branches of Purkinje cells are often thicken, distorted, or crowded (e, arrows), differently from PtAcacDMS-treated rats (f) showing a rich and fine Purkinje cell branching as in controls. *Treatment with dose of 10 *μ*g/g b.w.* At PD17, the dendrites of Purkinje cells in cisPt-treated rats spread within the entire ML (g), but some branches are lacking (g, arrows). After PtAcacDMS (h), the labelling evidences similar features as in controls. Magnification: 40x.

 The calcium binding protein calbindin is a specific marker of Purkinje neurons, especially for dendrite branches [85]. In comparison with control rats (Figures 3(a) and 3(d)), the acute effects of the treatment with cisPt (Figure 3(b)) showed Purkinje cells with shorter and less branched dendrites, but later, seven days after the treatment (Figure 3(e)), the tree of Purkinje neurons was expanded within the ML similarly to the controls (Figure 3(d)). These morphological changes are included in the neuroanatomical and functional alterations resulting from early postnatal cerebellar insults in rodents (for review, see [6]). CisPt effects are dose dependent; high dose (10 *μ*g/g b.w) did not further affect the growth of Purkinje neuron trees but seriously altered their morphology; the dendrites of Purkinje cells in cisPt-treated rats spread within the entire ML (Figure 3(g)), but some branches are lacking.

 Likewise cisPt (Figure 3(b)), PtAcacDMS slackened the growth of Purkinje cell trees at the early stage (Figure 3(c)) after the injection [74] as seen by calbindin immunoreaction. Later, differently from cisPt (Figure 3(e)), the treatment with the new platinum compound (Figure 3(f)) did not apparently affect the dendrite branching and extension of Purkinje neurons. Moreover, cisPt at higher dose (Figure 3(g)) deeply altered Purkinje cell growth, while after PtAcacDMS treatment, almost normal dendrites were observed (Figure 3(h)).

 GABA is the major inhibitory neurotransmitter in the cerebellum; it is present in Purkinje, basket, stellate, and Golgi cells. The functional labelling of Purkinje cell differentiation steps has been obtained by the enzyme of synthesis of GABA, that is, GAD, a marker also for synaptic sites [86, 87]. 

**Figure 4:**
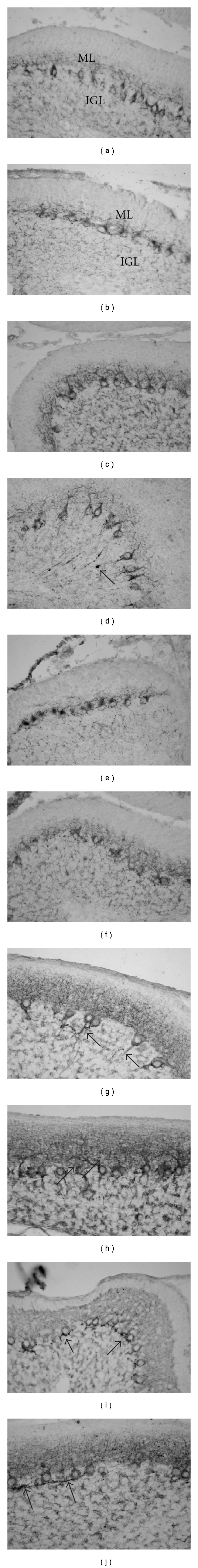
Purkinje cell differentiation and synaptogenesis: GAD67 immunoreaction.* Treatment with dose of 5* **μ*g/g b.w.* At PD11 (a)–(d), in control rats (a), GAD67 labelling is shown in the entire Purkinje neurons and the dot-like nerve terminals in the ML, and the inhibitory components (Golgi neuron axons) of the glomeruli in the IGL. After cisPt treatment, a marked delay in the Purkinje cell dendrite growth and a low number of punctuate nerve terminals in the ML are observed (b). The GAD67 labelling after PtAcacDMS displays almost normal patterns (c), except for presence of axonal swellings in the form of spheroids in some Purkinje cells (d, arrow). At PD17 (g, h), seven days after treatments, differently from cisPt (not shown), the GAD67 immunopositive baskets at the Purkinje cell axon hillock (arrows) are apparently normal (as in controls, g) after PtAcacDMS at low dose (h). *Treatment with dose of 10 *μ*g/g b.w.* At PD11 (e, f), the Purkinje cell differentiation is further delayed after cisPt (e), differently from that observed in PtAcacDMS rats (f). At PD17 (i, j), deeply altered baskets and lack of immunopositive nerve terminals in the ML are observed after cisPt (i); the treatment with PtAcacDMS induces several recurrent collaterals of ganglionic plexuses in some tracts of convolutions, but dot-like immunopositive terminals are present in the ML (j). Magnification: 40x.

 Immunocytochemistry for GAD67 shows labelling of the entire Purkinje neurons, the basket at the axon hillock of Purkinje neurons, the inhibitory nerve terminals in the ML and the inhibitory components (Golgi neuron axons) of the glomeruli in the IGL. All these structures, including the maturing basket, were visible in controls at PD11 (Figure 4(a)). CisPt treatment, at both doses, induced a marked delay in the Purkinje cell dendrite growth and low number of nerve terminals in the ML, (Figure 4(b) and 4(e)), while GAD67 labelling after PtAcacDMS displayed almost normal patterns (Figure 4(c) and 4(f)), except for the presence of axonal swellings in the form of spheroids in some Purkinje cells (Figure 4(d)). Seven days after treatments, at PD17, differently from cisPt (not shown), the GAD67 immunopositive basket at the Purkinje cell axon hillocks was apparently normal after PtAcacDMS at low dose (Figure 4(h)) in respect of controls (Figure 4(g)). Moreover, comparing with the high dose of cisPt that deeply altered the baskets and the immunopositivity of nerve terminals in the ML (Figure 4(i)), the PtAcacDMS induced several recurrent collaterals of ganglionic plexuses in some tracts of convolutions (Figure 4(j)).

 Agents that blocks, directly or indirectly, signalling information for further development of basic circuits disturb CNS development [2]. As previously reported, a single dose of cisPt affects several neural transmitters and their receptors [24–27]. In particular, the changes of the GABA synthesis enzyme (GAD) immunolabelling in the developing cerebellum offered the chance of evaluating the morphological and functional damages in the neural cortical circuits after cisPt, that is, Purkinje cell dendrite growth, basket structure at the Purkinje cell axon hillock, and presence and distribution of inhibitory synapses in the ML and IGL [23]. The new platinum compound induces few alterations of the GABA inhibitory cerebellum circuits. 

### 3.3. Brain-Blood Barrier

The developing brain is characterised by the absence or incomplete Brain-Blood Barrier (BBB). The development of BBB is a gradual process beginning *in utero* terminating around 21 days after birth in the rat [88]. Thus, some toxic agents that never enter the mature brain enter the developing brain freely. In the mature brain, platinum-based agents have the propensity to enter the dorsal root ganglia and peripheral nerves [21] but not the brain, due to poor penetration through BBB. 

 In our studies, we demonstrated the ability both of cisPt and PtAcacDMS to reach the developing brain tissue once injected [74]. Interestingly, the brain platinum content after PtAcacDMS administration was up to 4-fold higher than after cisPt. This might mean that the novel Pt complex is less bound to plasma proteins than cisPt and can cross the BBB more readily. In the developing cerebellum, when the treatment has been performed, the BBB cannot impede cisPt passage from the blood into the cerebral tissue. On the other hand, studies show that the administration of cisPt is associated with an increase in the BBB permeability further facilitating the passage of cisPt across the barrier [89, 90]. Neural and vascular lesions produced by this agent are manifested as foci of hemorrhagic necrosis and edema [89], in accordance with what we previously reported on cerebellar cortex, where disrupted or damaged blood vessel walls are visible in hemorrhagic areas after cisPt treatment [91]. 

 The presence of platinum in the brain tissue after PtAcacDMS administration fits in with data obtained from *in vitro* studies on HeLa and MCF-7 cells [46, 53]. Similarly to cisPt, cellular accumulation measurements showed that the accumulation of novel Pt compound is linearly correlated with the drug concentration. On the other hand, the kinetics of PtAcacDMS uptake is different from cisPt. PtAcacDMS concentration in the cells increases rapidly, and its cellular accumulation in HeLa and MCF-7 cells is, respectively, about 6 and 10 times higher than that of cisPt [46, 53]. Since it is known that the cytostatic effect is closely associated with the accumulation of platinum in the cell, this could represent an advantage for PtAcacDMS in-so-far as it would permit to use lower doses of this Pt complex reducing, at the same time, the risk of side effects and drug resistance. 

### 3.4. Recovery and Remodelling

Animal and human studies in the last two decades have demonstrated that plasticity reorganization occurs in the mammalian nervous system in response to peripheral and central injuries. The extent of plasticity changes is usually greater in the developing brain than in the adult brain although the adult brain is still capable of a considerable degree of reorganization (for review, see [92]).

 Recovery of proliferating activity has been reported after treatment with cytostatic drug [80], including cisPt [12] at late stages from injection. The regenerative capabilities of the EGL in the developing cerebellum [5] replaces partially cell loss, but remodelling events resulting in neuroanatomical and functional alterations are often required (for review, see [6]).

 In our schedule of cisPt treatment, we have reported both recovery of the neurogenetic processes in the EGL starting from a week after treatment and the persistence of alterations in Bergmann radial glia persisted [12], leading to ectopia of granule cells [12, 26]. This event has been largely described in the morphological alterations induced by X-ray irradiation during cerebellar histogenesis [93, 94]; ectopia of granule cells is considered a consequence of late neurogenetic inputs, and it is produced when the late descending granule cells stopped in the ML [93]. The ectopia of granule cells has deep consequences on proper development of cerebellar cortex cytoarchitecture (for review, see [6]). We provide evidence that remodelling of Purkinje cell dendrites, with reorientation of the main dendrite branches, and changes in the inhibitory control of compartments of Purkinje cell dendrites, occurs starting from a week after cisPt injection [26]; moreover, the elimination phase of climbing fibre synapses during Purkinje cell differentiation and the formation/competition of parallel and climbing fibres in the soma and the deep dendrite branches of Purkinje cells was altered or delayed [27].

 The studies carried out so far show that after PtAcacDMS treatment, no early apparent signs of reorganization in the cerebellar cortex architecture were found, at least for the markers studied till now. But, this is not a paradox since cell death and recovery, and their markers, of the proliferative activity were not drastically changed compared with controls; Purkinje cell dendrites displayed a less altered morphology, also at the higher dose (10 *μ*g/mg b.w.). Nevertheless, one day after injection, the presence of some Purkinje cells with axonal swellings in the form of spheroids has been observed; these alterations have been also described after intoxication with acrylamide [95]. These early alterations in the developing cerebellum, together with hypertrophy of ganglionic plexuses, observed later also after cisPt, may be considered signs of compensatory reaction to Purkinje cell axon injury and degeneration that means to start a program of axon regeneration in a permissive environment [96, 97]. This finding may be indicative of the presence of injured cells within the Purkinje population. Researches are in progress to evaluate possible persistent damages and recovery within this neuron population and cerebellar cortex architecture. 

## 4. Conclusions

PtAcacDMS induces less severe changes than cisPt on fundamental events of normal CNS development, that is, no significant apoptotic events, less altered granule cell migration, and Purkinje cell dendrite growth, pointing to a reduced neurotoxicity of this Pt complex. Moreover, the balance between Bcl2/Bax proteins favours the PtAcacDMS treated rats assuring normal features. The mild cytotoxic effects could be attributable to different subcellular targets of this compound as well as to a greater efficiency of cell repair system to recognize the drug-target adducts and to repair them. Together with the demonstrated antineoplastic effectiveness *in vitro* [46, 53], these results suggest that PtAcacDMS may be a potential alternative to cisPt indicating, at the same time, that the choice of cisPt analogues with new subcellular targets could be a strategy to prevent neurotoxicity induced by platinum compounds. Moreover, the ability of PtAcacDMS to exert a cytotoxic action on MCF-7 breast cancer cells, a human cell line relatively resistant to cisPt, could further promote the use of this Pt complex in cancer treatment to overcome resistance to chemotherapy, at least in case of drug resistance induced by mutations in the intrinsic apoptotic pathway. 

## Abbreviations

BBB: Blood-Brain Barrier
cisPt: Cisplatin
CNS: Central nervous system
EGL: External granular layer
IGL: Internal granular layer
ML: Molecular layer
PD: Postnatal day
PL: Purkinje cell layer
PtAcacDMS: [Pt(O,O′-acac)(*γ*-acac)(DMS)].
